# Fludarabine Inhibits Infection of Zika Virus, SFTS Phlebovirus, and Enterovirus A71

**DOI:** 10.3390/v13050774

**Published:** 2021-04-27

**Authors:** Chengfeng Gao, Chunxia Wen, Zhifeng Li, Shuhan Lin, Shu Gao, Haida Ding, Peng Zou, Zheng Xing, Yufeng Yu

**Affiliations:** 1 Jiangsu Key Laboratory of Molecular Medicine, Medical School, Nanjing University, Nanjing 210093, China; MG1735005@smail.nju.edu.cn (C.G.); chunxiawen@smail.nju.edu.cn (C.W.); linli_ltd@126.com (S.L.); 171232507@smail.nju.edu.cn (S.G.); Txhaidading@yeah.net (H.D.); 2 Jiangsu Provincial Center for Disease Control and Prevention, Department of Acute Infectious Diseases Control and Prevention, Nanjing 210009, China; andylizifeng@163.com; 3 Shanghai Public Health Clinical Center, Fudan University, Shanghai 201508, China; zoupeng@shphc.org.cn; 4 Department of Veterinary Biomedical Sciences, College of Veterinary Medicine, University of Minnesota at Twin Cities, Saint Paul, MN 55108, USA

**Keywords:** fludarabine, Zika virus, Severe fever with thrombocytopenia syndrome virus, Enterovirus A71, antiviral drugs

## Abstract

Viral infections are one of the leading causes in human mortality and disease. Broad-spectrum antiviral drugs are a powerful weapon against new and re-emerging viruses. However, viral resistance to existing broad-spectrum antivirals remains a challenge, which demands development of new broad-spectrum therapeutics. In this report, we showed that fludarabine, a fluorinated purine analogue, effectively inhibited infection of RNA viruses, including Zika virus, Severe fever with thrombocytopenia syndrome virus, and Enterovirus A71, with all IC_50_ values below 1 μM in Vero, BHK21, U251 MG, and HMC3 cells. We observed that fludarabine has shown cytotoxicity to these cells only at high doses indicating it could be safe for future clinical use if approved. In conclusion, this study suggests that fludarabine could be developed as a potential broad-spectrum anti-RNA virus therapeutic agent.

## 1. Introduction

Viruses cause about 75% of all human infectious diseases and have remained a threat to human lives and public health [1]. Effective antiviral treatments have been developed for some pathogens, such as human immunodeficiency virus (HIV), and Hepatitis C virus (HCV) [2,3]. However, for most new and re-emerging viruses, such as Zika virus (ZIKV), Enterovirus A71 (EV-A71) and Severe fever with thrombocytopenia syndrome virus (SFTSV), effective antiviral therapies are lacking. While development of specific viral vaccines is lagging, exploring safe and effective broad-spectrum antiviral therapies is in urgent need to fight against new and re-emerging viral infections.

High dependence of viral replication on host cells renders the host as the target of antiviral drugs. Interferons modulate host immune function to inhibit viral replication [4]. Chloroquine interferes with host signaling pathways and/or inactivates host kinases to exert antiviral effects [5,6]. Ciclosporin A binds cyclophilin A and inhibits the activity of peptidyl-prolyl cis/trans isomerases, resulting in broad-spectrum antiviral effects against HIV-1, HCV, and mouse cytomegalovirus, etc. [7]. Cycloguanil and its analogues target host dihydrofolate reductase and display excellent antiviral effect against influenza virus and respiratory syncytial virus [8]. It is noted that antiviral drugs targeting the host often differ in viral inhibitory activity in vitro and in vivo [9].

Drugs targeting viruses can specifically inhibit viral infection without affecting the host. Nucleoside analogues, targeting viral polymerases, account for more than half of the antiviral drugs currently on the market [10] and can be used as broad-spectrum antiviral drugs. Ribavirin inhibits both RNA and DNA viruses, including influenza A and B viruses, adenovirus, and HCV, etc. [10]. Remdesivir mainly inhibits RNA viruses that belong to Cilioviridae (Ebola virus, EBOV), Paramyxoviridae, and Coronaviridae [11,12,13]. Vidarabine has inhibitory activity against herpesvirus, poxvirus, some rhabdovirus, hepatotropic virus, and RNA tumor viruses [14]. Broad-spectrum antivirals are promising to be the first line of therapeutics against new and re-emerging viral epidemic [15]. However, a broad-spectrum antiviral drug does not necessarily work against all viruses. Remdesivir fails in clinical phase III trial for EBOV [16]. Its viability as a treatment for SARS-COV-2 is in controversy [17]. Viruses can tolerate drugs after being exposed to them for a period due to viral mutations [18]. Therefore, development of new and more effective broad-spectrum antiviral drugs will improve the ability to handle future outbreaks caused by emerging and re-emerging viruses.

**Figure 1:**
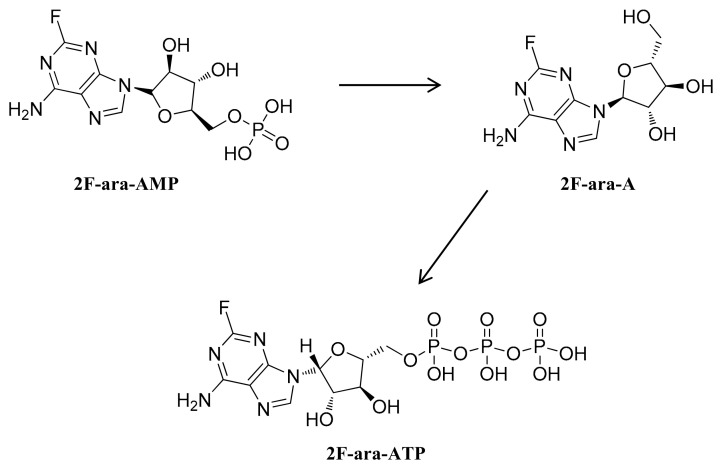
Activation of fludarabine. Fludarabine phosphate (2F-ara-AMP) is dephosphorylated to 9-P-D-arabinofuranosyl-2-fluoroadenine (Fludarabine, 2F-ara-A) in serum, which is taken up by cells. Upon entry into cells, 2F-ara-A is phosphorylated by deoxycytidine kinase to the pharmacologically active form, fludarabine triphosphate (2F-ara-ATP).

Fludarabine (2F-ara-A), a fluorinated purine analogue, is a chemotherapy drug for the treatment of chronic lymphocytic leukemia [19]. Because fludarabine is insoluble in water, fludarabine phosphate (2F-ara-AMP) is mostly used in clinic [19]. 2F-ara-AMP is dephosphorylated to 9-P-D-arabinofuranosyl-2-fluoroadenine (2F-ara-A) in serum, which is taken up by cells [20]. Upon entry into cells, 2F-ara-A is phosphorylated by deoxycytidine kinase to the pharmacologically active form, fludarabine triphosphate (2F-ara-ATP) (Figure 1), which further binds to nucleic acids and suppresses DNA synthesis by inhibiting ribonucleotide reductase and DNA polymerase [21,22]. In the present study, we found that fludarabine exhibited broad antiviral activity against emerging RNA viruses, including positive-stranded RNA viruses, ZIKV and EV-A71, and negative-stranded RNA virus SFTSV. We observed that fludarabine effectively inhibited viral RNA replication, protein expression and the cytopathic effect (CPE) caused by infection with ZIKV, EV-A71, or SFTSV. The inhibition of viruses was not cell-type dependent, as it showed potent antiviral activity in animal originated Vero and BHK21 as well as human astrocytes U251 MG and microglia HMC3 cells. In conclusion, our results suggest that fludarabine could potentially become a new broad-spectrum anti-RNA virus drug.

## 2. Materials and Methods

### 2.1. Cell Lines and Viruses

African green monkey kidney epithelial cells (Vero) and baby hamster kidney fibroblasts cells (BHK21) were kindly provided by Dr. Shibo Jiang in Fudan University. Human microglia HMC3 and human astrocytes U251 MG cells were purchased from the Shanghai Zhong Qiao Xin Zhou Biotechnology Co. Vero and BHK-21 cells were maintained in complete Dulbecco’s modified Eagle medium (DMEM, Gibco, CA, USA). HMC3 and U251 MG cells were cultured in Minimum Essential Medium (MEM, Gibco, CA, USA). Both the media were supplemented with 10% fetal bovine serum (FBS, ExCell Bio, Shanghai, China), 50 U/mL penicillin and 50 mg/mL of streptomycin (PS, Beyotime, Nanjing, China). All the cell lines were maintained at 37 °C supplemented with 5% CO_2_. Asian ZIKV strain SZ01 (GenBank number: KU866423) was isolated in serum from a Chinese patient who returned from Samoa [23]. ZIKV strain MR766 (#VR1838) was obtained from ATCC. SFTSV-A (JS2010-14), SFTSV-E (JS2014-16) [24] and EV-A71 (Fuyang strain, NCBI accession number: FJ439769) [25] were from Jiangsu Provincial Center for Disease Control and Prevention. Virus stock titers were quantified by standard plaque forming units (PFU) assay in BHK21 cells.

### 2.2. Quantitative Real-Time PCR (qRT-PCR) Assay

Fludarabine (MedChemExpress, Nanjing, China) was solubilized in dimethyl sulfoxide (DMSO; Thermo Fisher Scientific, Waltham, MA, USA) at 5 mM as a stock solution. To quantify viral RNA transcription in cells, cells were plated at a density of 10^5^ per well in 12-well plates overnight. Before infection, fludarabine, diluted to 1, 0.02, 0.04, and 0.008 μM or 5, 1.25, 0.31, and 0.08 μM, was mixed with the virus at a multiplicity of infection (MOI) of 0.1. After 2 h post infection (h p.i.), the cells were washed twice with PBS, and 2% FBS DMEM or MEM medium with fludarabine was added to cells. Total RNA was extracted at 24 h p.i. using TRIzol reagent (Thermo Fisher Scientific, Wilmington, DE, USA) for cDNA synthesis. cDNA was generated from 1 μg of total RNA using HiScript III RT SuperMix for qPCR (+gDNA wiper) (Vazyme, Nanjing, China). qRT-PCR was performed using TB Green^®^ Premix Ex Taq™ II (Tli RNaseH Plus) (Takara, Shiga, Japan) with ABI QuantStudio 5 or ABI Viia 7 PCR instrument (Thermo Fisher Scientific, Waltham, MA, USA) according to the manufacturer’s instructions. The primers used here are listed in Table 1.

### 2.3. Western Blot Analyses

Cells were seeded at a density of 2 × 10^5^ per well in six-well plates. After overnight, fludarabine diluted to the indicated concentration was mixed with 0.1 MOI of virus and added to the cells. Two hours later, the medium was replaced with fresh DMEM or MEM containing the indicated concentration of fludarabine and the cells were incubated at 37 °C for 36 h. The protein was lysed with RIPA Lysis Buffer (Beyotime, Shanghai, China) supplemented with protease and phosphatase inhibitors (Roche, Basel, Switzerland). SFTSV NP, ZIKV E, EV-A71 VP1 protein, and cellular β-actin protein (loading control) were detected using the anti-NP SFTSV mouse antibody (Cambridge Biologics, Suzhou, China), anti-ZIKV E rabbit antibody (Biodragon-Immunotech, Beijing, China), anti-VP1 EV-A71 mouse antibody (Cell Signaling Technology, Boston, MA, USA), and anti-beta actin mouse antibody (Proteintech, Wuhan, China), respectively. HRP conjugated goat anti-mouse IgG (H + L) and goat anti-rabbit IgG (H + L) (Proteintech, Wuhan, China) were used as the secondary antibody.

### 2.4. Immunofluorescence Assay

Vero cells were seeded onto 24-well chambered glass slides at a density of 2 × 10^4^ cells and slides were allowed to incubate overnight. The cells were infected with ZIKV SZ01 strain at 0.1 MOI for 2 h. After the inoculum was removed, the cells were incubated with medium containing different concentrations of fludarabine or DMSO. After 24 h p.i., the medium was removed and the cells were fixed for 30 min with paraformaldehyde followed by permeabilisation for 15 min with 0.1% Triton X-100 in PBS. After 3% BSA blocking for 40 min, viral dsRNA was immunostained with mouse anti-dsRNA J2 antibody (SCICONS, Sizlaku, Hungary) and Alexa Fluor 488-labelled donkey anti-mouse IgG (ThermoFisher Scientific, Wilmington, DE, USA). Cell nuclei were stained by 4′,6-diamidino-2-phenylindole (DAPI). Data were collected by laser scanning confocal microscopy (FV3000, Olympus, Tokyo, Japan).

### 2.5. Cytotoxicity Assay

A cell counting kit 8 (CCK8, Vazyme, Nanjing, China) assay was used to assess cytotoxicity of fludarabine in the cells according to the manufacturer’s instructions. Briefly, cells were seeded onto 96-well plates with 1 × 10^4^ cells per well for 12 h. Serial dilutions of fludarabine (diluted in DMEM or MEM with 2% FBS) and 1% DMSO were added to the medium, which were incubated for 48 h. After adding 10 μL of CCK8 working solution per well for 2 h, the absorbance value (OD) of each well was measured at 450 nm by a microplate reader (BioTek, Winooski, VT, USA).

### 2.6. Time-of-Addition Studies

For the pre-infection assay, Vero cells were treated with 0.5 μM fludarabine for 2 h before infection. For the co-infection assay, 0.1 MOI of virus and 0.5 μM fludarabine were added to Vero cells at the same time. For the post-infection assay, Vero cells were infected with 0.1 MOI of virus for 2 h, followed by replacement of the medium with 0.5 μM fludarabine added at specified time points (2, 8, 16, 24, 36 h p.i.). At 48 h p.i., Vero cells were collected for RNA extraction to measure viral RNA transcription by qRT-PCR assay.

### 2.7. Statistical Analysis

The concentration of fludarabine that inhibits virus replication by 50% (IC_50_) as well as the concentration of fludarabine that reduces host cell metabolism by 50% (CC_50_) was derived. All data were analyzed by GraphPad Prism 8 software (San Diego, CA, USA). Two-tailed Student’s *t* test was applied for comparison of the differences between two groups. Data are presented as mean ± SD; *, *p* < 0.05; **, *p* < 0.01; ***, *p* < 0.001; ns, no significance.

## 3. Results

### 3.1. Fludarabine Inhibited Infection of ZIKV

**Figure 2:**
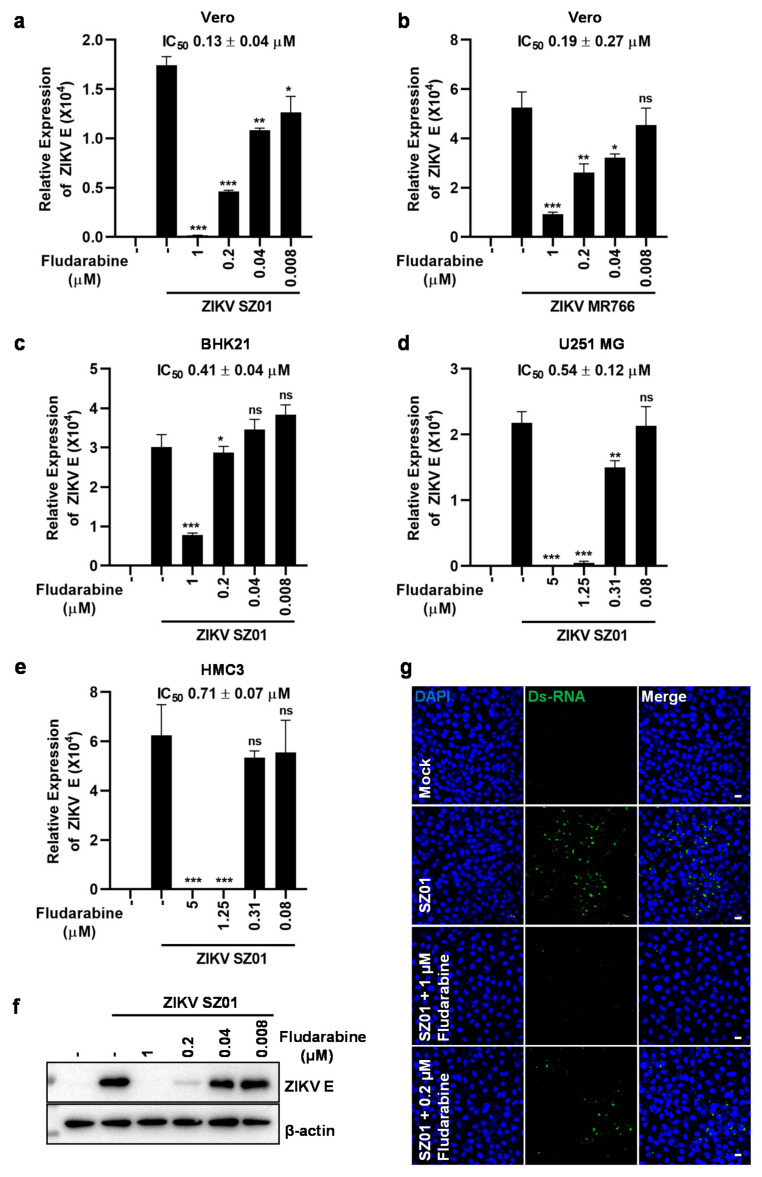
Fludarabine inhibited infection of ZIKV. (**a**,**b**) Inhibitory effect of fludarabine on ZIKV SZ01 and MR766 infection in Vero cells. (**c**–**e**) Inhibitory effect of fludarabine against ZIKV SZ01 infection in BHK21, U251 MG and HMC3 cells, respectively. Fludarabine, diluted to 1, 0.2, 0.04, and 0.008 μM or 5, 1.25, 0.31, and 0.08 μM, was mixed with 0.1 MOI of ZIKV SZ01 or ZIKV MR766. After the mixture was inoculated to cells for 2 h, the mixture was discarded and the cells were washed twice with PBS. The cell cultures were supplemented with DMEM or MEM (2% FBS) containing fludarabine of the same concentration as mentioned above. Total RNA was prepared 24 h later to measure ZIKV E gene transcription by qRT-PCR assay. The differences between infected-groups without and with treatment of fludarabine were evaluated by two-tailed Student’s *t* test. Data are presented as mean ± SD; *, *p* < 0.05; **, *p* < 0.01; ***, *p* < 0.001; ns, no significance; ‒, no fludarabine was added. (**f**) Decrease of viral protein in ZIKV-infected cells treated with fludarabine. The expression of ZIKV SZ01 E protein was measured by western blot analysis after the Vero cells were treated for 36 h as mentioned above. ‒, no fludarabine was added. (**g**) Anti-ZIKV activity of fludarabine was tested using immunofluorescence analysis. After the Vero cells were treated for 24 h as mentioned above, ZIKV SZ01 dsRNA was detected by immunostaining with J2 anti-dsRNA monoclonal antibody (green). Cell nuclei were stained by 40, 6-diamidino-2-pheny-lindole (DAPI, blue). Scale bars, 20 μm.

To evaluate the antiviral efficacy on different genotypes of ZIKV, fludarabine was serially diluted in DMEM and mixed with 0.1 MOI of ZIKV strain SZ01 or MR766. The mixtures were added to Vero cells and incubated for 2 h. After the medium was replaced by fresh DMEM containing fludarabine, the cells were cultured for 24 h. Finally, the transcription of ZIKV E gene was measured by qRT-PCR. As shown in Figure 2a,b, fludarabine dose-dependently inhibited viral replication in Vero cells infected with both SZ01 and MR766 strains with IC_50_ values of 0.13 ± 0.04 μM for SZ01 and 0.19 ± 0.27 μM for MR766, respectively. Similarly, fludarabine inhibited ZIKV SZ01 infection in a dose dependent manner with IC_50_ value of 0.41 ± 0.04 μM in BHK-21 cells (Figure 2c), 0.54 ± 0.12 μM in U251 MG cells (Figure 2d), and 0.71 ± 0.07 μM in HMC3 cells (Figure 2e). These results suggested that fludarabine effectively inhibited ZIKV infection in a variety of cell types, including neural cells. These data were confirmed at the level of viral proteins in infected cells as we examined the expression of ZIKV SZ01 E protein by western blot assay. As shown in Figure 2f, ZIKV SZ01 E protein expression was dose-dependently inhibited by fludarabine. Subsequently, we examined the inhibitory effect of fludarabine on viral dsRNA formation in Vero cells by immunofluorescence. We observed in Figure 2g that the dsRNA formation of ZIKV SZ01 visibly decreased in the fludarabine group compared with the control group and 1 μM of fludarabine completely inhibited the formation of ZIKV SZ01 dsRNA. These results suggest that fludarabine could inhibit ZIKV RNA replication and protein expression.

### 3.2. Fludarabine Inhibited Infection of SFTSV

**Figure 3:**
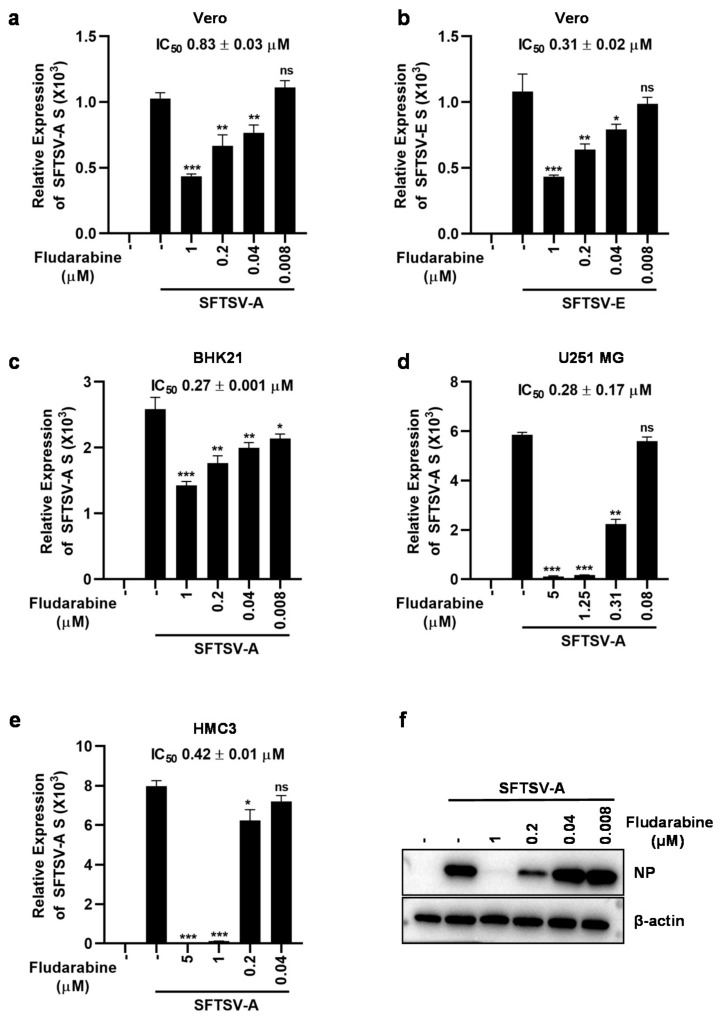
Fludarabine inhibited infection of SFTSV. (**a**–**e**) Inhibitory effect of fludarabine on SFTSV-A or SFTSV-E infection in Vero, BHK21, U251 MG and HMC3 cells. Fludarabine, diluted to 1, 0.2, 0.04, and 0.008 μM or 5, 1.25, 0.31, and 0.08 μM, was mixed with 0.1 MOI of SFTSV-A or SFTSV-E. After the mixture was inoculated to cells for 2 h, the mixture was discarded and the cells were washed twice with PBS. The cell cultures were supplemented with DMEM or MEM (2% FBS) containing fludarabine of the same concentration as mentioned above. Total RNA was prepared 24 h later to measure SFTSV S gene transcripts by qRT-PCR assay. The differences between infected-groups without and with treatment of fludarabine were evaluated by two-tailed Student’s *t* test. Data are presented as mean ± SD; *, *p* < 0.05; **, *p* < 0.01; ***, *p* < 0.001; ns, no significance; ‒, no fludarabine was added. (**f**) Anti-SFTSV activity of fludarabine was evaluated by western blot analysis. The expression of SFTSV-A nucleoprotein (NP) was measured by western blot analysis after the Vero cells were treated for 36 h as mentioned above. ‒, no fludarabine was added.

To detect the inhibitory activity of fludarabine on SFTSV, we infected Vero cells with SFTSV-A (JS2010-14) or SFTSV-E (JS2014-16), which are in separate clades evolutionarily, after incubation with fludarabine and detected viral Small (S) gene transcription after 24 h p.i. As shown in Figure 3a,b, fludarabine inhibited SFTVS replication in a concentration-dependent manner with IC_50_ values of 0.83 ± 0.03 μM for the SFTSV-A strain (Figure 3a) and 0.31 ± 0.02 μM for the SFTSV-E strain (Figure 3b), indicating that fludarabine inhibition of SFTSV was not viral strain dependent. The results in BHK21 cells were similar to those in Vero cells, with an IC_50_ of 0.27 ± 0.001 μM for the SFTSV-A strain (Figure 3c). We observed that SFTSV replicated well in both U251 MG and HMC3 cells. To analyze whether fludarabine also had a protective effect against SFTSV infection in neuronal cells, we tested the antiviral activity of fludarabine in U251 MG and HMC3 cells. The results showed that fludarabine dose-dependently inhibited SFTSV-A replication in both U251 MG and HMC3 cells with an IC_50_ of 0.28 ± 0.17 μM in U251 MG cells (Figure 3d) and 0.42 ± 0.01 μM in HMC3 cells (Figure 3e). These results were further confirmed in the infected cells that viral nucleoprotein (NP) expression was obviously inhibited by fludarabine (Figure 3f) in Vero cells, consistent with the qRT-PCR results, indicating that fludarabine could effectively inhibit SFTSV infection in various cell types.

### 3.3. Fludarabine Inhibited Infection of EV-A71

**Figure 4:**
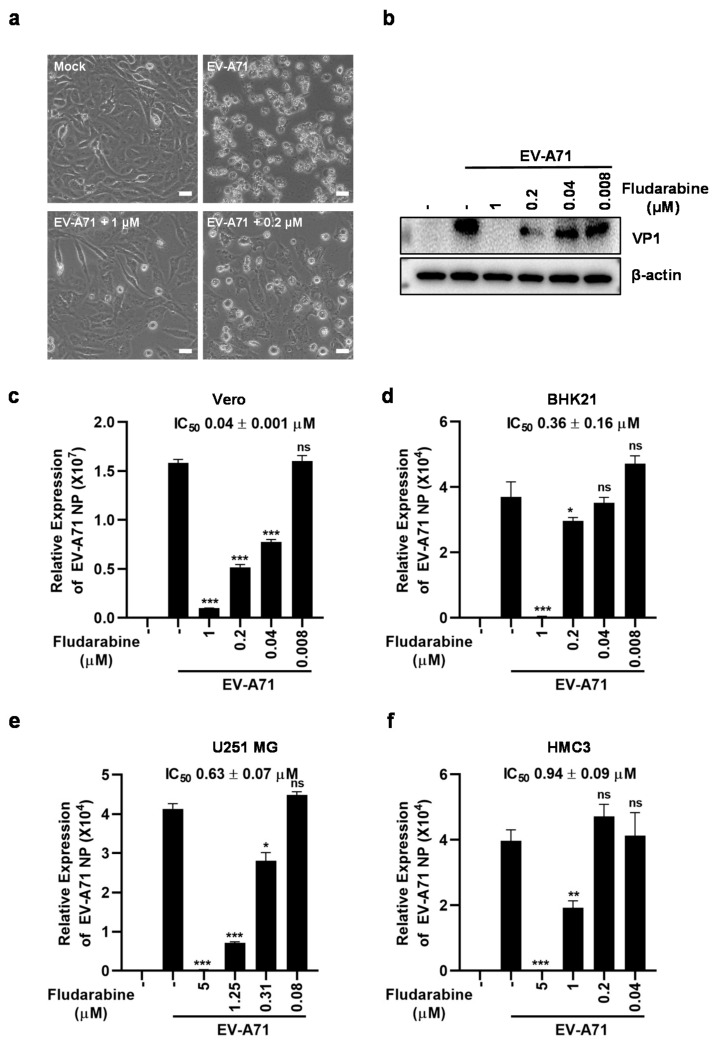
Fludarabine inhibited infection of EV-A71. (**a**) Fludarabine reduced EV-A71-induced cytopathic effect (CPE) in Vero cells. Scale bars, 20 μm. (**b**) Fludarabine inhibited EV-A71 viral capsid protein 1 (VP1) expression in Vero cells. ‒, no fludarabine was added. (**c–f**) Antiviral activity of fludarabine against EV-A71 in Vero, BHK21, U251 MG, and HMC3 cells. Serially diluted fludarabine was mixed with 0.1 MOI of EV-A71. After the mixture was inoculated to cells for 2 h, the mixture was discarded and the cells were washed twice with PBS. The cell cultures were supplemented with DMEM or MEM (2% FBS) containing fludarabine. After Vero cells were treated as mentioned above for 36 h, CPE was observed under a microscope (**a**) and EV-A71 VP1 protein expression was determined by western blot analysis (**b**). Total RNA was prepared at 24 h p.i. to measure EV-A71 nucleoprotein (NP) gene transcripts by qRT-PCR assay (**c**–**f**). The differences between infected-groups without and with treatment of fludarabine were evaluated by two-tailed Student’s *t* test. Data are presented as mean ± SD; *, *p* < 0.05; **, *p* < 0.01; ***, *p* < 0.001; ns, no significance; ‒, no fludarabine was added.

To investigate whether fludarabine inhibited EV-A71 infection, we infected Vero cells with 0.1 MOI of EV-A71 after co-incubation with different concentrations of fludarabine. Thirty-six h p.i., the cytopathic effect (CPE) and viral capsid protein 1 (VP1) expression in infected cells were observed. As shown in Figure 4a, cells in the Mock group were intact with healthy morphology, while cells in the EV-A71-infected group became shrunken, rounded and detached. The treatment with fludarabine could visibly reduce CPE caused by EV-A71 infection, especially at a concentration of 1 μM. We next determined the inhibitory effect of fludarabine on EV-A71 replication in Vero cells. As shown in Figure 4c, fludarabine exhibited concentration-dependently inhibition of EV-A71 replication as shown by decreased transcription of viral NP gene measured by qRT-PCR with an IC_50_ of 0.04 ± 0.001 μM. The expression of EV-A71 VP1 protein was also dose-dependently reduced by fludarabine in Vero cells (Figure 4b) detected by western blot analysis. The results in BHK21 cells were similar to those in Vero cells with an IC_50_ of 0.36 ± 0.16 μM (Figure 4d). Further, we tested whether fludarabine could be against EV-A71 infection in neural cells. As shown in Figure 4e,f, fludarabine dose-dependently inhibited EV-A71 replication in both U251 MG and HMC3 cells with an IC_50_ of 0.63 ± 0.07 μM in U251 MG cells and 0.94 ± 0.09 μM in HMC3 cells. The above results indicated that fludarabine could be an effective inhibitor of EV-A71 infection in both neural and non-neural cells.

### 3.4. Fludarabine May Suppress Viral RNA Replication

**Figure 5:**
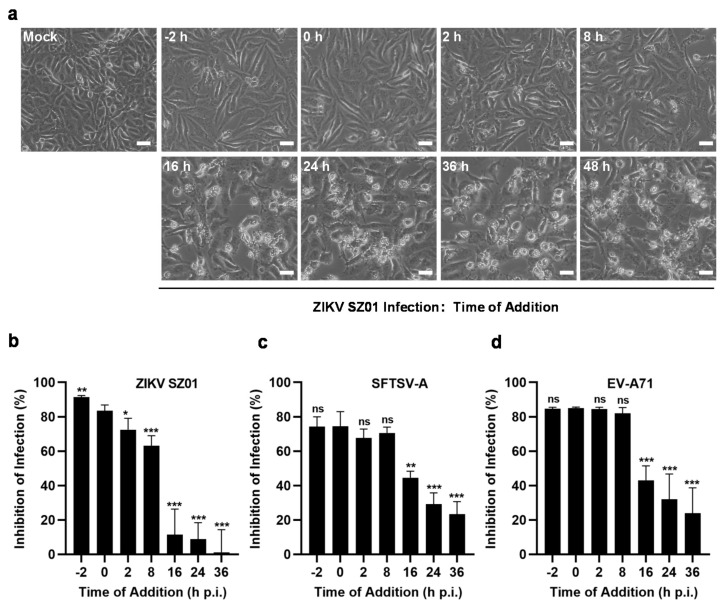
Fludarabine inhibited the viral replication in the first 8 h of viral infection. (**a**) ZIKV SZ01-induced cytopathic effect (CPE) on Vero cells with fludarabine addition at different time points p.i. Scale bars, 20 μm. (**b**–**d**) Time of addition experiment of fludarabine against ZIKV SZ01, SFTSV-A, and EV-A71. Fludarabine diluted to 0.5 μM was added to Vero cells 2 h prior to virus infection or at 0, 2, 8, 16, 24, or 36 h p.i. Virus replication was determined by qRT-PCR assay at 48 h p.i. ZIKV SZ01-induced CPE was observed under a microscope (**a**). The differences between groups of fludarabine added at 0 h and other time points were evaluated by two-tailed Student’s *t* test. Data are presented as mean ± SD; *, *p* < 0.05; **, *p* < 0.01; ***, *p* < 0.001; ns, no significance.

The above findings demonstrated that fludarabine inhibited the infection of ZIKV, SFTSV, and EV-A71 in Vero, BHK21, U251 MG, and HMC3 cells in a dose-dependent manner, suggesting that fludarabine could inhibit RNA viruses regardless of cell types. Next, we conducted a time-of-addition study to explore which stage of the viral cycle fludarabine works at. Fludarabine was added at different time points after ZIKV SZ01 infection started. CPE was observed under a microscope at 48 h p.i. As shown in Figure 5a, the ZIKV SZ01-caused CPE were clearly reduced when fludarabine was added within the first 8 h than that after 16 h p.i. We next examined the antiviral effect of fludarabine which was added at different time points p.i. by qRT-PCR. The results showed that fludarabine pre-treatment for 2 h suppressed more than 91.43%, 74.25%, and 84.68% of infections with ZIKV SZ01 (Figure 5b), SFTSV-A (Figure 5c), and EV-A71 (Figure 5d), respectively. The addition of fludarabine at 8 h p.i. still suppressed more than 55.59%, 70.55% and 81.98% of infections with ZIKV SZ01 (Figure 5b), SFTSV-A (Figure 5c), and EV-A71 (Figure 5d), respectively. The inhibition of viral infections was significantly reduced when fludarabine was added at 16 h p.i. In conclusion, fludarabine mainly inhibits viral proliferation within the first 8 h p.i., suggesting that fludarabine may have impact on viral RNA replication.

### 3.5. Cytotoxicity of Fludarabine on Various Cells

**Figure 6:**
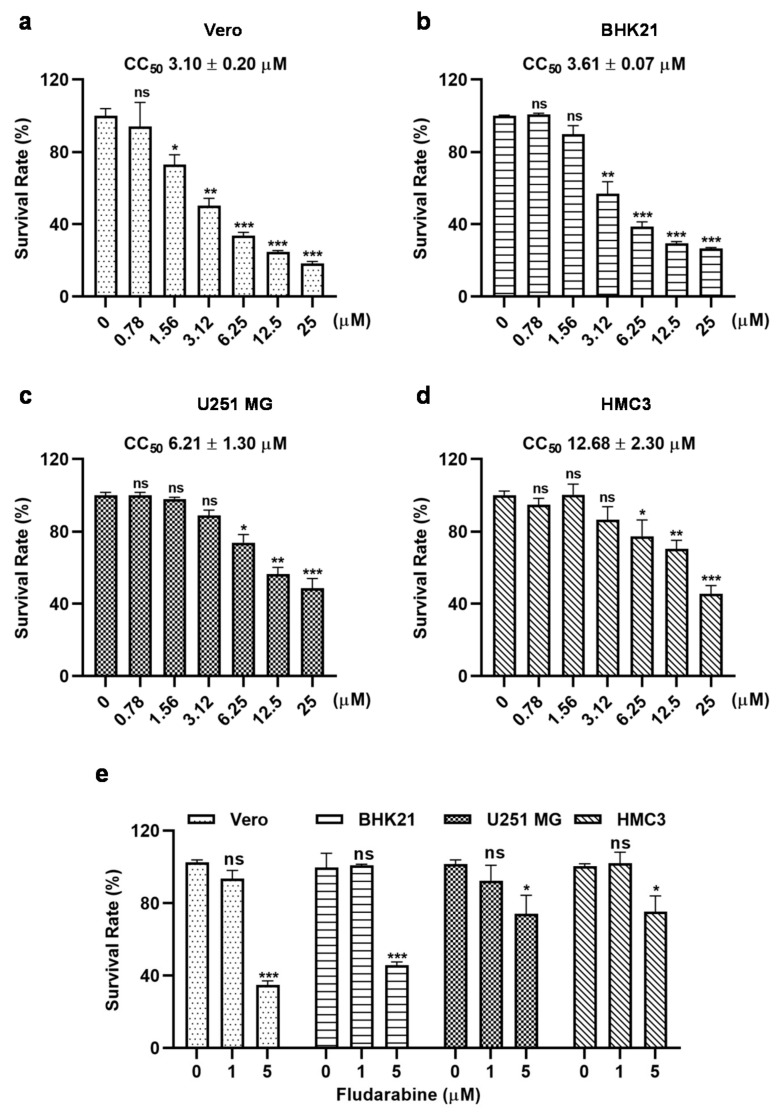
Cytotoxicity of fludarabine. (**a**–**e**) Cytotoxicity of fludarabine in Vero, BHK21, U251 MG, and HMC3 cells. Cells were incubated with the indicated concentrations of fludarabine for 48 h, followed by measuring cell viability using CCK8 assay. The differences between groups without and with fludarabine at different concentrations were evaluated by two-tailed Student’s *t* test. Data are presented as mean ± SD; *, *p* < 0.05; **, *p* < 0.01; ***, *p* < 0.001; ns, no significance.

To determine the cytotoxicity of fludarabine, we performed CCK8 analysis in Vero, BHK21, U251 MG and HMC3 cells. As shown in Figure 6a–d, the viability of all cells decreased in a fludarabine dose-dependent manner, and the 50% cytotoxic concentration (CC_50_) was higher in U251 MG and HMC3 cells than in Vero and BHK21 cells, with a CC_50_ of 6.21 ± 1.30 μM in U251 MG cells, 12.68 ± 2.30 μM in HMC3 cells, 3.10 ± 0.20 μM in Vero cells, and 3.61 ± 0.07 μM in BHK21 cells, respectively. Since the maximum concentration of fludarabine used in our antiviral experiments was 1 μM in Vero and BHK21 cells and 5 μM in U251 MG and HMC3 cells, we analyzed the cytotoxicity of the fludarabine at the above concentrations. As shown in Figure 6e, Vero and BHK21 cells did not show significant cytotoxicity when exposed to fludarabine at 1 μM. Though U251 MG and HMC3 cells exhibited statistically significant cytotoxicity at 5 μM of fludarabine, they remained 74.14% and 75.20% of survival rate (Figure 6e), respectively. Therefore, we assess that the antiviral activity exhibited by fludarabine on ZIKV, SFTSV, and EV-A71 in our study was not caused by its cytotoxicity.

## 4. Discussion

In this report, we demonstrate that fludarabine effectively inhibited infections of ZIKV (SZ01 and MR766 strain), SFTSV (Chinese genotype A and Japanese genotype E), and EV-A71, regardless of cell types (Figure 2, Figure 3 and Figure 4), suggesting that fludarabine is a promising compound to be a broad-spectrum antiviral. Notably, fludarabine showed inhibition of viral infection in human astrocytes U251 MG and human microglial HMC3 cells, suggesting that fludarabine may have some neuroprotective effects, which we will further verify by animal experiments. Our results show fludarabine functioned mainly within the first 8 h of viral infection (Figure 5), indicating that fludarabine may inhibit viral RNA replication. As for the mechanism for fludarabine to inhibit viral RNA replication, our hypothesis is that fludarabine triphosphate (2F-ara-ATP), the active form of fludarabine, may compete with naturally occurring nucleotides for incorporation into viral RNA, which could interfere with the function of the viral RNA-dependent RNA polymerase and even terminate viral RNA synthesis.

It was reported that Fludarabine also inhibits phosphorylation of STAT1 [26]. Since this activity does not counteract its antiviral activity, fludarabine could even be a good choice for treating some emerging viral infections including H5N1 and SARS-CoV-2, in which cytokine storm is critical to pathogenesis. We are currently experimenting antiviral activities of fludarabine on viruses in this category.

Vidarabine, a broad-spectrum antiviral drug, has a mechanism of inhibition on nucleic acid synthesis, similar to fludarabine [22]. However, it is easily deaminated and inactivated by adenosine deaminase (ADA) to become ara-hypoxanthine [14]. Unlike vidarabine, fludarabine possesses some tolerance to ADA [27]. Thus, fludarabine as a small molecule drug has a long half-life and good bioavailability in vivo. The mean (±SD) elimination half-life of 2F-ara-A in patients with B-cell chronic lymphocytic leukemia was 11.3 (±4.0) h after oral dosage (25 mg/m^2^, *n* = 14), and 9.7 (±2.0) h after intravenous dosage (40 mg/m^2^, *n* = 42) [28]. Estimated mean oral bioavailability of 2F-ara-A was 58% [28]. 2F-ara-A persisted for about 6 h at plasma concentration above 120 ± 20 ng/mL (0.42 ± 0.07 μM) as shown in a previous study [28], which is the average IC_50_ value for fludarabine against the three types of RNA viruses in our study. Therefore, fludarabine is expected to reach blood levels sufficient to suppress viral replication. We estimate that fludarabine will display strong antiviral activity in vivo which will be tested in suitable animal models, such as A129 mice [29] or newborn mice [30].

Fludarabine exhibited some cytotoxicity in our study (Figure 6), which is disadvantageous commonly seen among nucleoside analogues. In clinical trials, fludarabine was found to reduce renal function [31], suppress bone marrow formation and poison neural systems at high doses [32]. Severe fever with thrombocytopenia syndrome often presents with acute kidney and liver injury or multiple organ failure due to hypercytokinemia and disseminated intravascular coagulation. Therefore, fludarabine may exhibit more severe cytotoxicity and adverse effects if used in the SFTS patients at the later stage of their disease course, or in other patients with existing conditions including organ failure. However, numerous studies have shown that orally taken fludarabine phosphate 2F-ara-AMP has a reliable record of safety [28] and studies on further reducing its toxicity in clinical use are still underway and proven valuable. The pharmacokinetic properties, antiviral activity and safety of the drug can be improved by masking the phosphoryl oxygen groups with ProTides (phenyl and benzyl esters of salicyl alcohol), introducing S-Acyl-2-thioethyl protecting group, and conjugating amino acid and peptide, etc. [10,33]. Trimethyl derivatives of fludarabine have shown strong anti-DENV activity with no toxicity at concentration up to 20 μM [34], suggesting that reducing toxicity of fludarabine is achievable.

## 5. Conclusions

In summary, fludarabine is shown to exhibit a broad-spectrum antiviral activity against ZIKV, EV-A71, and SFTSV across positive- and negative-stranded RNA viruses in variety of cell types and has a potential to be a potent antiviral compound against some emerging viral infections.

## Figures and Tables

**Table 1:** Primers used in this study.

Primer Name	Sequence
ZIKV E-F	GGGTTGATGTTGTCTTGGAACAT
ZIKV E-R	AGGCTTCACCTTGTGTTGGG
EV71 NP-F	CGCCCAAGGTTGTGACACGATT
EV71 NP-R	ACTATGCCGACGACGCCATGTT
SFTSV-A S-F	GCAAGATGACCAACACAGTATGGTT
SFTSV-A S-R	CCACTAGGCCACCTAAGAGCA
SFTSV-E S-F	GGGTCCCTGAAGGAGTTGTAAA
SFTSV-E S-R	GGCAAGATGCCTTCACCAA
Vero GAPDH-F	TCAACAGCGACACCCACTC
Vero GAPDH-R	CTTCCTCTTGTGCTCTTGCT
BHK21 GAPDH-F	TCGGAGTGAACGGATTTGG
BHK21 GAPDH-R	TTCTCAGCCTTGACTGTGCC
Human β-actin-F	AAGGAGAAGCTGTGCTACGTCGC
Human β-actin-R	AGACAGCACTGTGTTGGCGTACA

## Data Availability

The data presented in this study are available on request from the corresponding author.
